# Corneal Limbal Microenvironment Can Induce Transdifferentiation of Hair Follicle Stem Cells into Corneal Epithelial-like Cells

**DOI:** 10.1634/stemcells.2008-0721

**Published:** 2009-03

**Authors:** Ewa Anna Blazejewska, Ursula Schlötzer-Schrehardt, Matthias Zenkel, Björn Bachmann, Erik Chankiewitz, Christina Jacobi, Friedrich E Kruse

**Affiliations:** Department of Ophthalmology, University of Erlangen-NurembergErlangen, Germany

**Keywords:** Adult stem cells, Cornea, Hair follicle, Transdifferentiation, Epithelial lineages, Limbal stem cell niche

## Abstract

The aim of this study was to investigate the transdifferentiation potential of murine vibrissa hair follicle (HF) stem cells into corneal epithelial-like cells through modulation by corneal- or limbus-specific microenvironmental factors. Adult epithelial stem cells were isolated from the HF bulge region by mechanical dissection or fluorescence-activated cell sorting using antibodies to α6 integrin, enriched by clonal expansion, and subcultivated on various extracellular matrices (type IV collagen, laminin-1, laminin-5, fibronectin) and in different conditioned media derived from central and peripheral corneal fibroblasts, limbal stromal fibroblasts, and 3T3 fibroblasts. Cellular phenotype and differentiation were evaluated by light and electron microscopy, real-time reverse transcription-polymerase chain reaction, immunocytochemistry, and Western blotting, using antibodies against putative stem cell markers (K15, α6 integrin) and differentiation markers characteristic for corneal epithelium (K12, Pax6) or epidermis (K10). Using laminin-5, a major component of the corneo-limbal basement membrane zone, and conditioned medium from limbal stromal fibroblasts, clonally enriched HF stem and progenitor cells adhered rapidly and formed regularly arranged stratified cell sheets. Conditioned medium derived from limbal fibroblasts markedly upregulated expression of cornea-specific K12 and Pax6 on the mRNA and protein level, whereas expression of the epidermal keratinocyte marker K10 was strongly downregulated. These findings suggest that adult HF epithelial stem cells are capable of differentiating into corneal epithelial-like cells in vitro when exposed to a limbus-specific microenvironment. Therefore, the HF may be an easily accessible alternative therapeutic source of autologous adult stem cells for replacement of the corneal epithelium and restoration of visual function in patients with ocular surface disorders.

## INTRODUCTION

The cornea of the eye is composed of three layers—an outer stratified, rapidly regenerating epithelium, the underlying stroma, and an inner single-cell layered endothelium. Homeostasis of corneal epithelial cells is an important prerequisite not only for the integrity of the ocular surface but also for corneal transparency and visual function. The continuous renewal of the corneal epithelium is provided by a population of stem cells located in the basal epithelium of the transitional zone between cornea and conjunctiva, known as the limbus [1]. Stem cell maintenance and function are controlled by various intrinsic and extrinsic factors provided by a unique local microenvironment or niche [2]. Common components of such niches are signaling molecules and growth factors from neighboring cells as well as specialized extracellular matrices, which regulate cell phenotype and behavior through cell-matrix interactions [3, 4]. Limbal stem cells and their progeny, the transient amplifying cells, reside within small clusters in the basal epithelium in close spatial relationship with specific basement membrane and matrix components [5] as well as with stromal fibroblasts and blood vessels providing increased levels of growth and survival factors [2].

Damage to or dysfunction of the limbal stem cell population due to different inherited or acquired conditions results in limbal stem cell deficiency, which has severe consequences for ocular surface integrity and visual function and may lead to functional blindness. One successfully used therapeutic strategy for ocular surface reconstruction is the transplantation of autologous epithelial cell sheets engineered from limbal epithelial cells expanded in vitro using appropriate delivery systems, such as amniotic membrane or fibrin gels [6, 7]. This approach, however, usually requires a limbal biopsy from the contralateral healthy eye and is therefore not applicable in patients with bilateral total limbal stem cell deficiency. In these cases, allogeneic limbal epithelium, harvested either from living related donors or from cadaveric donor eyes, may be used for transplantation in combination with prolonged systemic immunosuppressive therapy. This approach has a very low success rate long term, as compared with autologous cells, and immunosuppressants are not always well tolerated by patients. Therefore, present research activities focus on the evaluation of alternative autologous stem cell sources for ex vivo culture and transplantation avoiding the risk of immune-mediated rejection and the need for immunosuppression. Oral mucosal epithelium has attracted much attention as an autologous epithelial stem cell source, and the transplantation of cultivated oral mucosal epithelial cell sheets has provided favorable early results in patients with bilateral stem cell deficiency [8, 9]. Long-term outcomes were, however, less satisfactory, mostly due to a relatively high rate of peripheral corneal neovascularization [10]. Therefore, alternative stem cell-based therapeutic strategies for ocular surface repair and reconstruction are still required.

The discovery of various, not yet completely defined, stem cell populations in the epithelial and mesenchymal compartment of the hair follicle (HF) of adult mice and humans [11] has encouraged research into utilizing the HF as a readily accessible source of adult multipotent stem cells for regenerative medicine. In addition to stem cells in the HF dermal papilla or connective tissue sheath, which are capable of differentiating into hematopoietic, adipogenic, osteogenic, chondrogenic, myogenic, and neurogenic lineages [12–15], epithelial stem cells residing in the bulge region of the outer root sheath can differentiate into hair follicles, sebaceous gland, and epidermis after injury [11, 16, 17]. This particular keratinocyte stem cell population has been characterized by expression of cytokeratins K5, K15, K17, and K19 [18–20]. Bulge-specific cell surface markers, such as α6 integrin, CD34, and CD200, have been used to isolate and purify stem cells from the HF bulge region [21–24].

The HF bulge area as a reservoir of epithelial stem cells might represent a useful, hitherto not explored, autologous source of adult stem cells in therapeutic approaches for ocular surface reconstruction and other disorders of keratinocyte lineage. This study seeks to explore the basic capacity of adult HF epithelial stem cells to transdifferentiate into corneal epithelial-like cells in a murine model. In order to direct stem cell differentiation into a corneal epithelial phenotype, appropriate corneal- and limbus-specific signals were provided in vitro using both specific extracellular matrix components and conditioned media harvested from fibroblasts isolated from the central and peripheral cornea as well as from the limbal stroma. The findings provide evidence that adult murine HF keratinocyte stem cells are capable of differentiating into corneal epithelial-like cells in vitro when exposed to a limbus-specific microenvironment.

## MATERIALS AND METHODS

### Preparation of Culture Media

The basic culture medium for murine HF epithelial cells was prepared containing three parts Dulbecco's modified Eagle's medium (DMEM) and one part Ham's F12 medium (0.4 mM Ca^2+^) (HyClone, Logan, UT, http://www.hyclone.com) supplemented with 10% fetal calf serum (FCS) (Invitrogen, Karlsruhe, Germany, http://www.invitrogen.com), 10 ng/ml human epidermal growth factor (Chemicon, Temecula, CA, http://www.chemicon. com), 500 mg/l L-glutamine (Invitrogen), 0.2% bovine pituitary extract, 0.18 μg/ml hydrocortisone, 5 μg/ml insulin, and 5 μg/ml transferrin (Tebu-Bio, Paris, France, http://www.tebu-bio.com) as well as 10,000 U/ml penicillin, 10,000 μg/ml streptomycin, and 25 μg/ml amphotericin B (Invitrogen). Culture medium for 3T3 murine fibroblasts contained DMEM supplemented with 10% FCS (Invitrogen) and 10,000 units/ml penicillin, 10,000 μg/ml streptomycin, and 25 μg/ml amphotericin B (Invitrogen).

### Coating of Chamber Slides with Extracellular Matrix Components

Glass chamber slides (Nunc, Rochester, NY, http://www.nuncbrand.com) were coated with various matrix components, including type IV collagen (40 μg/ml), fibronectin (20 μg/ml), laminin-1 (100 μg/ml) (BD Biosciences, San Diego, http://www.bdbiosciences.com), and laminin-5 (10 μg/ml) (Chemicon) by incubation for 1 hour at room temperature. Afterwards, the slides were briefly washed with phosphate-buffered saline (PBS) and dried under a laminar flow bench before seeding of clonal cells. To gain the appropriate concentrations of the coating substrates, the commercially acquired stock solutions were diluted using unconditioned serum-free culture medium.

### Conditioning of Culture Medium with Human Corneal and Limbal Fibroblasts

Corneal and limbal fibroblasts were isolated from human donor corneas not suitable for transplantation. Corneal buttons were dissected into central, peripheral, and limbal regions. Incubation of tissue specimens in 2.4 U/ml dispase II (Roche, Basel, Switzerland, http://www.roche-applied-science.com) for 1.5 hours at 37°C was followed by mechanical separation of the corneal epithelium from the underlying stroma. The remaining connective tissue was cut into small pieces and placed in 2 mg/ml collagenase A (Roche) overnight at 37°C. After digestion, the fibroblasts were seeded into cell culture flasks (BD Falcon, San Diego, http://www.bdbiosciences.com) and cultivated for 24 hours in DMEM:Ham's F12 (3:1) medium (1.2 mM Ca^2+^) (Hyclone). After cell adhesion and spreading, the medium was changed to DMEM:Ham's F12 (3:1) (0.4 mM Ca^2+^) for another 48 hours and then collected, filtered through a 40-μm cell strainer (Millipore, Billerica, MA, http://www.millipore.com), and stored at −80°C until use. The harvested media were designated conditioned media (CM). Only the first three passages (P1, P2, and P3) of freshly isolated and cultivated corneal and limbal fibroblasts were used for production of CM. In addition, CM from 3T3 fibroblasts was prepared accordingly and used as a control, because primary cultures of murine keratinocytes require fibroblast CM for long-term culture [25]. For all experiments, CM were added to the normal culture media at a ratio of 1:1.

### Preparation of Vibrissae

Three- to five-week old C57Bl/6 mouse pups (Charles River Laboratories International, Inc., Kisslegg, Germany, http://www.criver.com) were sacrificed by methohexital injection and subsequent cervical dislocation. The upper lip containing the pads with the vibrissae was dissected and its inner surface was exposed. The subcutaneous fat and the connective tissue were carefully removed and the vibrissae were then pulled away from the pad using fine forceps under a dissecting microscope. The HF together with its surrounding collagen capsule was cut into three fragments (Fig. 2A, 2B). The first transversal cut was made just below the sebaceous gland. The second cut was made at the site of insertion of the nerve fibers, which is proximal to the site of inferior enlargement of the outer root sheath. Each fragment was then separately transferred into a 35-mm dish containing 1 ml of collagenase A/dispase II (1 mg/ml; Roche) and incubated for 30 minutes at 37°C. After complete digestion of the collagen capsule, the HF fragments were transferred into fresh cell culture dishes containing 0.05% trypsin and incubated for about 1.5 hours until cell dissociation was completed.

### Fluorescence-Activated Cell Sorting

Freshly isolated HF cells were suspended in PBS-EDTA (5 mM) and stained with α6 integrin-fluorescein isothiocyanate (FITC)-conjugated CD49f antibody diluted 1:100 (BD Biosciences) for 30 minutes on ice. Following three washing steps in PBS-EDTA, α6 integrin-positive and α6 integrin-negative cells were isolated using a MoFlo cell sorter (Cytomation Bioinstruments GmbH, Freiburg, Germany). Sorted cells were collected as two living cell populations and enriched by clonal expansion on a 3T3 feeder layer.

### Clonal Expansion and Colony-Forming Efficiency Assay

Clonal expansion of HF stem cells was performed on a mitomycin C-treated 3T3 feeder layer using three different approaches: (a) bulge explant outgrowth, (b) single-cell suspensions obtained by mechanical dissection and enzymatic digestion, and (c) single-cell suspensions obtained by fluorescence-activated cell sorting (FACS). For single-cell approaches, equal numbers of isolated HF cells (1 × 10^3^ cells/cm^2^) were plated onto mitomycin C-treated 3T3 feeder cells in six-well culture plates. After 14 days of culture, the feeder layer was removed using Versene (Invitrogen) and stem cell colonies were visualized by staining with 2% rhodamine B (Sigma-Aldrich, Munich, Germany, http://www.sigmaaldrich.com). The colony-forming efficiency (CFE) was calculated using the following formula: number of colonies formed/number of cells plated × 100%.

### Subculture of Clonal Cells Under Various Environmental Conditions

Epithelial stem cells from the HF bulge region were clonally enriched on a 3T3 feeder cell layer as described above. After 14 days of cultivation, the feeder layer was removed using Versene (Invitrogen) and clonal cells were trypsinized (0.25% trypsin-EDTA) (Invitrogen) for 15 minutes at 37°C. Clonal cells were seeded at a density of 1 × 10^5^ cells/cm^2^ onto glass chamber slides or fibrin gels coated with different matrix components and were subcultivated for 8-16 days in fibroblast CM obtained from the central cornea, the peripheral cornea, and the limbus. CM obtained from 3T3 fibroblasts served as a control. Culture conditions were switched from low-calcium medium (0.4 mM Ca^2+^) to high-calcium medium (1.2 mM Ca^2+^) after 7 days in order to promote differentiation.

### Light and Transmission Electron Microscopy

For light and electron microscopic analysis, clonal cells were subcultured on fibrin gels under various environmental conditions. The fibrin gels were prepared by dissolving fibrinogen and thrombin stock solutions (Tissucol; Baxter BioPharma Solutions, Halle, Germany, http://www.baxterbiopharmasolutions.com) in 1.1% NaCl and 1 mM CaCl_2_ to a final concentration of 10 mg/ml fibrinogen and 3 IU/ml thrombin [26]. The gels were fixed in either 4% paraformaldehyde or 2% glutaraldehyde in 0.1 M phosphate buffer, dehydrated, and embedded in paraffin or epoxy resin, respectively, according to standard protocols. Paraffin sections were stained with hematoxylin and eosin, and ultrathin sections were stained with uranyl acetate-lead citrate and examined with an electron microscope (EM 906E; Carl Zeiss AG, Oberkochen, Germany, http://www.zeiss.de).

### Immunohistochemistry

Immunofluorescence labeling experiments were performed on cryosections of mouse eyes and HF, on stem cell colonies expanded on a 3T3 feeder layer, and on subcultivated cell sheets. For immunolabeling of cell sheets, clonal cells were plated onto either glass chamber slides (Nunc) or fibrin gels and cultivated under various environmental conditions, such as different matrix coatings and different CM. After 2 weeks of cultivation, the cells were fixed with 70% ethanol for 10 minutes, washed, and permeabilized with 0.1% Triton X-100 in PBS for 10 minutes. After blocking with 10% normal serum (Dako, Glostrup, Denmark, http://www.dakousa.com), the cells were stained with mouse-specific primary antibodies against K10, K12 (1:50; Santa Cruz, Biotechnology Inc., Santa Cruz, CA, http://www.scbt.com), K3/12, K15 (1:100; Abcam, Cambridge, U.K., http://www.abcam.com), Pax6 (1:100; Abcam), and α6 integrin (CD49f, 1:100; Chemicon) for 2 hours at room temperature. Antibody binding was detected by Alexa Fluor 488-conjugated secondary antibodies (Invitrogen-Molecular Probes, Karlsruhe, Germany, http://www.invitrogen.com) and nuclear counterstaining was performed with propidium iodide or 4′,6′-diamino-2-phenylindole (Sigma-Aldrich). Slides were washed three times in PBS and coverslipped with Vectashield (Vector Laboratories, Ltd., Peterborough, U.K., http://www.vectorlabs.com) prior to evaluation with a fluorescence microscope (Zeiss). In negative control experiments, the primary antibody was replaced by PBS or equimolar concentrations of an irrelevant primary antibody.

### Real-Time Reverse Transcription-Polymerase Chain Reaction

Total RNA was isolated (RNeasy kit; Quiagen, Hilden, Germany, http://www1.qiagen.com) from cells expanded under various environmental conditions as well as from mouse epidermis and corneal epithelium. First-strand cDNA synthesis from 500 ng of total RNA and quantitative real-time reverse transcription-polymerase chain reaction (RT-PCR) was performed with a thermal cycler and software (MyIQ; Bio-Rad Laboratories GmbH, Munich, Germany, http://www.bio-rad.com) as previously described [27]. PCRs were run in duplicate using the following program: 95°C for 3 minutes, and 40 cycles of 95°C for 30 seconds, 62°C for 30 seconds, and 72°C for 30 seconds. Exon-spanning primers (MWG Biotech AG, Ebersberg, Germany, http://www.mwg-biotech.com) for mouse K10 (NM_010660.2; 5′: AGTGGTACGAGAAGCATGGCAAC, 3′: CATTGTCAGTTGTCAGGGTGAGG), K12 (NM_010661.2; 5′: GCAGGAGATAGTGAATGGCGAAGTG, 3′: TGCTAACAAGACCCAACCTGCATAG), Pax6 (NM_013627 5′: AGGGCCAAATGGAGAAGAGAAG, 3′: CCAACATGGAACCTGATGTGAA), and β-actin (NM_007393; 5′: GTGACGTTGACATCCGTAAAGACC, 3′: GGAGCCAGAGCAGTAATCTCCTTC) were designed by means of Primer three software (available at http://www-genome.wi.mit.edu/genome_software/other/primer3.html). For quantification, serially diluted standard curves of plasmid-cloned cDNA were run in parallel, and amplification specificity was checked using melting curve and sequence analyses (Prism 3100 DNA sequencer; Applied Biosystems Deutschland GmbH, Darmstadt, Germany, http://www3.appliedbiosystems.com). For normalization of gene expression levels, mRNA ratios relative to the housekeeping gene β-*actin* were calculated.

### Western Blot Analysis

Total protein was isolated from cells expanded under various environmental conditions using RIPA buffer (150 mM NaCl, 1% Nonidet P-40, 0.5% deoxycholate, 0.1% SDS, 50 mM Tris pH 8, 10 μg/ml Aprotinin [Roche]). Ten micrograms total protein was separated by SDS-PAGE under reducing conditions, and immunoblot analyses were performed using antibodies against mouse K12 (1:100; Santa Cruz), K10 (1:1,000; Abcam), Pax6 (1:1,000; Abcam), and β-actin (1:1,000; Abcam) and evaluated as previously described [28].

### Statistics

Data are presented as the mean ± standard deviation. Statistical evaluation of significant differences between different assays was performed with the Mann-Whitney test for nonparametric analysis. *p* < .05 was considered statistically significant.

## RESULTS

### Expression of Stem Cell and Differentiation Markers in Mouse HF and Cornea

**Figure 1:**
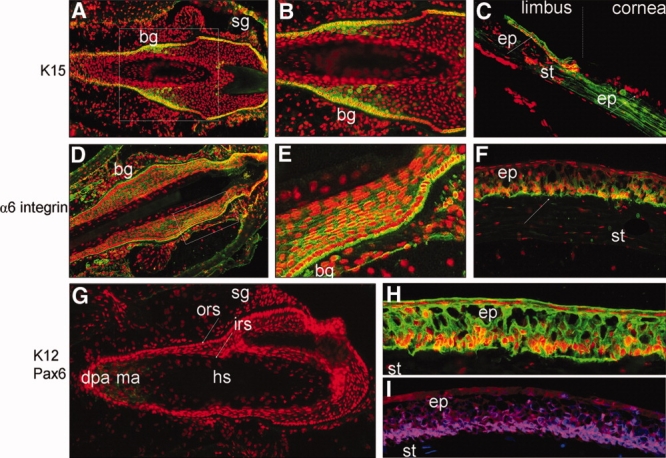
Expression of stem cell and differentiation markers in murine hair follicle (HF) and cornea. **(A, B):** Expression of K15, a putative epithelial stem and progenitor cell marker, in the HF bulge and basal cells. **(C):** Selective expression of K15 in the basal cells of limbal epithelium. **(D, E):** Expression of α6 integrin, a putative stem and progenitor cell marker, in HF bulge and basal cells. **(F):** Expression of α6 integrin in the basal cells of the corneal epithelium. **(G):** Lack of expression of K12 and Pax6 in the murine vibrissae HF. **(H):** Expression of K12 (green fluorescence) in murine corneal epithelium. **(I):** Expression of Pax6 (red fluorescence) in the cell nuclei of corneal epithelium throughout all the epithelial cell layers. Nuclear staining was performed with propidium iodide (red) or 4′,6-diamidino-2-phenylindole (blue). Magnification: 200× **(E, I, H)**, 100× **(B, C, F, G)**, 40× **(A, D)**. Abbreviations: bg, bulge; dpa, dermal papilla; ep, epithelium; hs, hair shaft; irs, inner root sheath; ma, matrix; ors, outer root sheath; sg, sebaceous gland; st, stroma.

Expression patterns of putative stem cell and differentiation markers were compared by immunohistochemistry on frozen sections of murine HF and cornea. The putative stem cell markers K15 and α6 integrin, which have been previously reported to be expressed in the murine HF bulge region at high levels [29, 30], were confirmed to be strongly expressed in both bulge cells and basal cells of the HF outer root sheath (Fig. 1A, 1B, 1D, 1E). Whereas K15 could be immunolocalized to the cytoplasm of bulge and basal cells (Fig. 1A, 1B), α6 integrin was more widely expressed and localized to cell membranes of bulge and basal cells (Fig. 1D, 1E). Both markers were also observed in the murine corneo-limbal region, showing distinct K15 staining of basal cells in the limbal epithelium (Fig. 1C) and α6 integrin staining of basal cells in the corneal epithelium (Fig. 1F). K10, a marker of terminally differentiated epidermal keratinocytes, was expressed in neither HF nor corneal epithelial cells (data not shown). K12, a marker of differentiated corneal epithelial cells, was found to be expressed exclusively in the corneal epithelium (Fig. 1H) and not in the HF (Fig. 1G). Pax6, a transcription factor for K12, was found to be expressed mainly in the basal cells of the corneal epithelium (Fig. 1I) and was also lacking in the HF (Fig. 1G). These different expression patterns were confirmed using real-time RT-PCR and Western blotting (data not shown). Together, these observations indicate important parallels regarding stem cell markers and differences regarding differentiation markers between the HF and the corneo-limbal region in the mouse model.

### Isolation and Clonal Enrichment of HF Bulge Cells

To isolate HF bulge cells and to enrich colony-forming epithelial stem cells, two different approaches were performed and compared regarding CFE and phenotype of clonal cells: (a) mechanical dissection and enzymatic dissociation of the bulge cells and (b) FACS using an antibody against α6 integrin.

**Figure 2:**
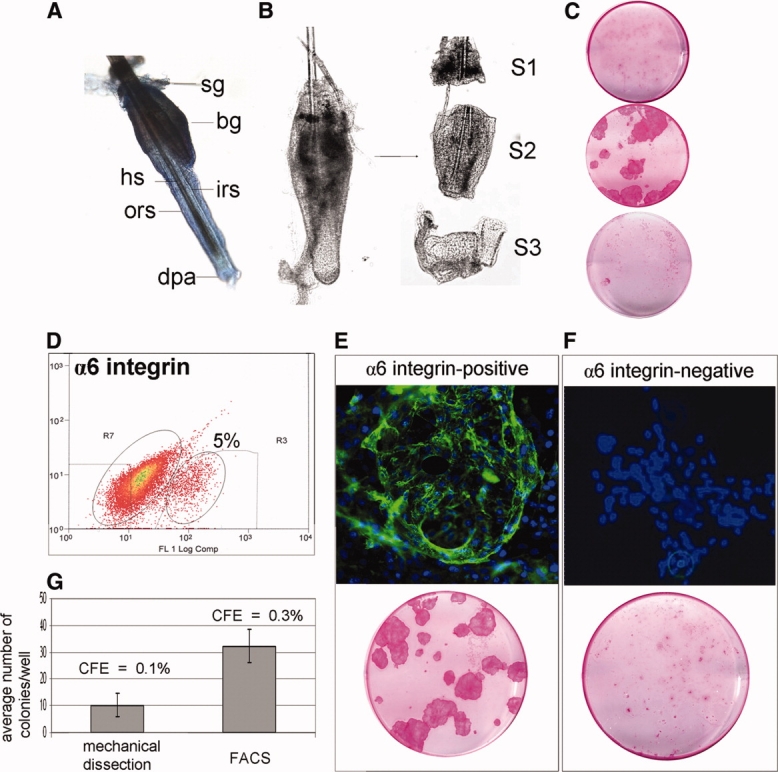
Isolation of hair follicle (HF) bulge cells. **(A):** Isolated HF after enzymatic digestion of the collagen capsule. **(B):** Dissection of the isolated HF into three fragments: S1, sebaceous gland-containing section; S2, bulge-containing section; S3, dermal papilla and matrix-containing section. **(C):** Colony-forming assay performed with cells derived from S1, S2, and S3 on a 3T3 feeder cell layer; rhodamin B staining after 2 weeks of culture. **(D):** Fluorescence-activated cell sorting (FACS) of isolated HF cells using an antibody to α6 integrin; the α6 integrin-positive cell population accounts for about 5% of the total cells**. (E, F):** Clonogenic capacity of α6 integrin-positive **(E)** and α6 integrin-negative **(F)** cell populations obtained by FACS. **(G):** Calculation of the colony-forming efficiency (CFE) of an α6 integrin-positive cell population after cell sorting in comparison with the CFE of the bulge cells after mechanical dissection. The bar chart demonstrates the mean number of colonies ± standard deviation from five experiments. Magnification: 100× **(E, F)**, 40× **(A, B)**. Abbreviations: bg, bulge; dpa, dermal papilla; hs, hair shaft; irs, inner root sheath; ors, outer root sheath; sg, sebaceous gland.

**Figure 3:**
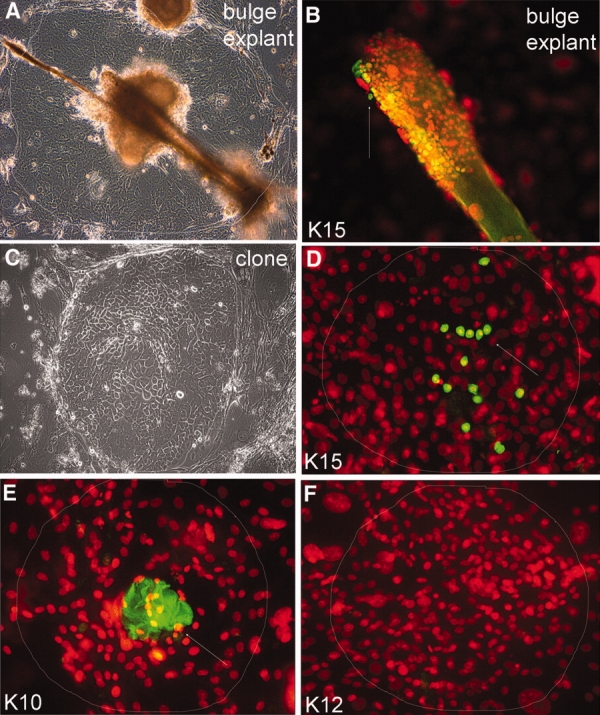
Clonal enrichment of hair follicle (HF) bulge cells. **(A):** Cellular outgrowth of HF bulge explants after 7-10 days of culture on a 3T3 feeder layer. **(B):** Expression of K15, an epithelial stem cell marker, in the outgrowing cells (arrow) of a bulge tissue explant. **(C):** Light microscopy of a holoclone obtained by either mechanical dissection or fluorescence-activated cell sorting on a 3T3 feeder layer. **(D):** K15-positive basal cells (arrow) are present in the culture of a holoclone. **(E):** K10-positive cells (arrow) are present in the culture of a holoclone, confined to a suprabasal layer of the central focus of stratification. **(F):** Lack of K12 expression within a holoclone. Nuclear staining was performed with propidium iodide (red). Magnification: 100× **(D–F)**, 40× **(A–C)**.

For mechanical separation and enzymatic digestion, vibrissae follicles (*n* = 50), measuring about 3 mm in length and comprising the epithelial root sheaths, the bulge, the sebaceous gland, and the dermal papilla (Fig. 2A), were dissected and carefully cut into three fragments (Fig. 2B). The section containing the sebaceous gland was designated S1, the bulge-containing section was designated S2, and the remaining part containing matrix and papilla was designated S3. To evaluate the clonogenic potential of the different portions, single-cell suspensions obtained from each section were plated onto a 3T3 feeder layer, cultivated for 14 days in calcium-low (0.4 mM Ca^2+^) medium, and then fixed and stained with 2% rhodamine B. Whereas cells isolated from regions S1 and S3 were not clonogenic, cells isolated from the bulge region S2 gave rise to large colonies measuring up to 10 mm in diameter with smooth perimeters, termed holoclones [31] (Fig. 2C). The CFE, which is considered to correlate with the number of stem cells present in the S2 cell population, was calculated to be 0.1% (Fig. 2G). Clonal cells could be serially cultivated up to six passages before they revealed phenotypic signs of senescence. These observations confirm previous reports, that clonogenic cells with high proliferative capacity, a typical characteristic of stem cells, are segregated in the bulge region of the murine hair follicle [32]. Consistently, explant cultures revealed cellular outgrowth and colony formation from the bulge region after 7-10 days (Fig. 3A). The outgrowing cells were immunopositive for the putative stem cell marker K15 (Fig. 3B).

In a second approach, freshly isolated HF cells were sorted by FACS using an FITC-conjugated antibody against α6 integrin (Fig. 2D). The resulting populations of α6 integrin-positive cells, representing about 5% of the total cell population, and α6 integrin-negative cells were seeded at a definite clonal density on a 3T3 feeder layer. After 2 weeks of culture, keratinocyte colonies were compared regarding CFE and immunopositivity for α6 integrin. The α6 integrin-positive cell population exhibited a significantly higher clonogenic capacity, with formation of holoclones, than the α6 integrin-negative cell population, producing a few paraclones only (Fig. 2E, 2F). The CFE of the α6 integrin-positive cell population was calculated to be 0.3% (Fig. 2G).

Phenotypic characterization of holoclones formed by either mechanical dissection or FACS showed their composition to be small, roundish, densely packed cells independent of the method of cell isolation (Fig. 3C). By immunocytochemistry, a few K15-positive cells, supposed to represent putative stem or progenitor cells, were present in the center of each holoclone (Fig. 3D). Most holoclones revealed a central focus of stratification with suprabasal cells expressing K10, typical of epidermal differentiation (Fig. 3E). The cornea-specific marker K12 was not expressed within the colonies (Fig. 3F). The proportion of K10-positive cells within the holoclones could be increased by elevating the calcium concentration (1.2 mM Ca^2+^) of the culture medium (data not shown).

These findings indicate that both mechanical dissection and FACS using α6 integrin antibodies followed by clonal expansion are effective methods to isolate and enrich epithelial stem and progenitor cell populations from the murine HF bulge region.

### Effect of Environmental Conditions on Growth and Differentiation of HF Stem Cells

**Figure 4:**
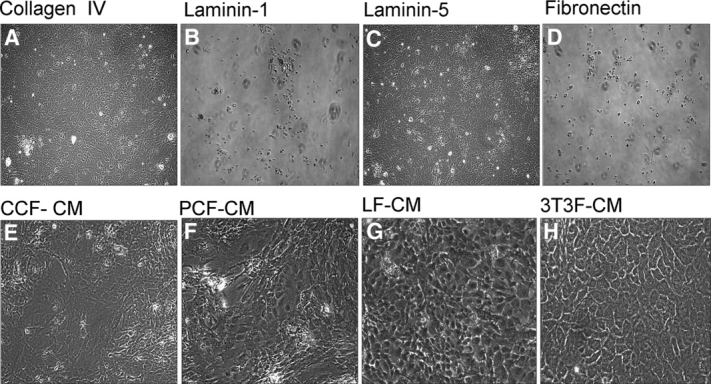
Effect of various culture conditions (matrices and conditioned media [CM]) on growth of clonally enriched, subcultivated hair follicle stem cells. **(A–D):** Light microscopic appearance of clonal cells subcultivated on type IV collagen **(A)**, laminin-1 **(B)**, laminin-5 **(C)**, and fibronectin **(D)**.Cultivation on type IV collagen and laminin-5 resulted in formation of regular epitheloid cell layers **(A, C)**, whereas laminin-1 and fibronectin adversely affected cell adhesion and growth **(B, D)**. **(E–H):** Light microscopic appearance of clonal cells subcultivated on laminin-5 in various CM from central corneal fibroblasts (CCF-CM) **(E)**, peripheral corneal fibroblasts (PCF-CM) **(F)**, limbal fibroblasts (LF-CM) **(G)**, and 3T3 fibroblasts (3T3F-CM) **(H)**. LF-CM and 3T3F-CM induced formation of regularly arranged, epitheloid cell sheets **(G, H)**, whereas CCF-CM and PCF-CM resulted in rather irregular growth patterns of cells **(E, F)**. Magnification: 100× **(A–D)**, 200× **(E–H)**.

Previous studies implicated type IV collagen, laminin-1, laminin-5, and fibronectin as major components of the basement membrane zone of corneal and limbal epithelia [5]. Clonal cells subcultivated on these matrix components showed significant differences in their adhesion properties. Subcultivation on type IV collagen and laminin-5 resulted in rapid cell adhesion and growth with about 90% of cells becoming adherent within 30 minutes after seeding. Both matrices supported the formation of regularly arranged monolayers of cuboid cells in a comparable manner (Fig. 4A, 4C). In contrast, laminin-1 and fibronectin adversely affected cell adhesion and growth resulting in about 10% of cells becoming attached to the substrates without formation of a confluent monolayer (Fig. 4B, 4D).

For further experiments, laminin-5 was selected as a substrate, because it represents a more specific component of the corneo-limbal basement membrane zone than the rather ubiquitous basement membrane component type IV collagen. Clonal cells subcultivated on laminin-5 in various CM for 8-10 days in calcium-high (1.2 mM Ca^2+^) medium showed significant differences in their growth patterns. Subcultivation in CM obtained from central and peripheral corneal fibroblasts resulted in rather irregular growth patterns, revealing partly enlarged and flattened and partly elongated cells (Fig. 4E, 4F). Using CM from limbal fibroblasts, clonal cells formed confluent, regularly arranged, cobble stone-like cell sheets composed of small cuboid cells (Fig. 4G), similar to cells grown in CM from 3T3 fibroblasts (Fig. 4H).

**Figure 5:**
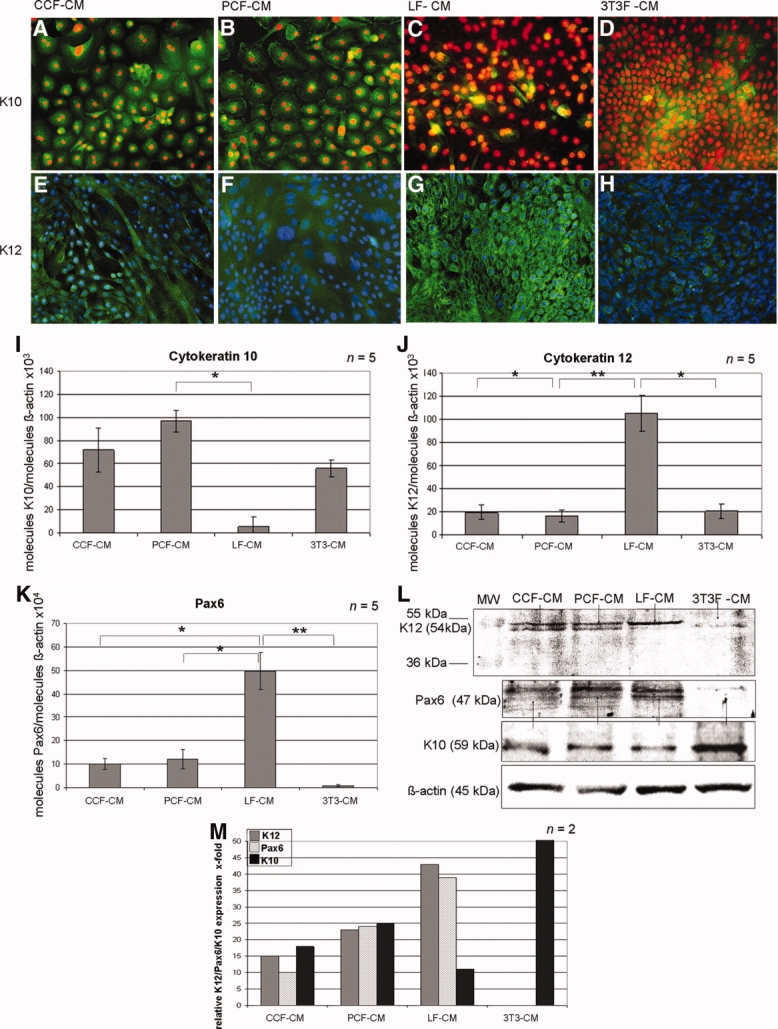
Effect of various conditioned media (CM) on differentiation of clonally enriched hair follicle stem cells subcultivated on laminin-5. **(A–D):** Expression of K10, an epidermal differentiation marker, in clonal cells exposed to CM from central corneal fibroblasts (CCF-CM) **(A)**, peripheral corneal fibroblasts (PCF-CM) **(B)**, limbal fibroblasts (LF-CM) **(C)**, and 3T3 fibroblasts (3T3F-CM) **(D)**. **(E–H)**: Expression of K12, a corneal differentiation marker, in clonal cells exposed to CCF-CM **(E)**, PCF-CM **(F)**, LF-CM **(G)**, and 3T3F-CM **(H)**. The lowest expression of K10 **(C)** and the highest expression of K12 **(G)** was observed in cells cultured in LF-CM. Nuclear staining was performed with propidium iodide (red) or 4′,6-diamidino-2-phenylindole (blue). Magnification: 100×. **(I–K):** Quantitative determination of K10, K12, and Pax6 mRNA expression levels of clonal cells cultured in various CM using real-time reverse transcription-polymerase chain reaction technology. The expression level was normalized against β-actin expression. The lowest expression of K10 **(I)** and the highest expression of K12 **(J)** and Pax6 **(K)** were observed in cells cultured in LF-CM. Statistical significance was assessed using the Mann-Whitney test for nonparametric analysis (*p < .05, **p < .005). **(L, M):** Determination of K12, K10, and Pax6 protein levels (arrows) in cells cultured in different CM by Western blot analysis based on a β-actin loading control. **(L):** Representative Western blot of cells cultured in CCF-CM, PCF-CM, LF-CM, and 3T3F-CM; MW, molecular weight marker. **(M):** Densitometric analysis of specific immunoreactive bands. Data were normalized to β-actin and represent the mean of two independent experiments.

The effect of different CM on K10 and K12 protein expression was analyzed by immunohistochemistry of clonal cells subcultivated in laminin-5 coated chamber slides. As expected, both markers could be immunolocalized to cytoplasmic filaments, but the proportion of K10- and K12-immunopositive cells showed significant differences depending on the CM used. Compared with 3T3 CM, CM obtained from central and peripheral corneal fibroblasts produced markedly higher proportions of K10-positive cells, whereas CM derived from limbal fibroblasts produced only a few K10-positive cells (Fig. 5A–5D). In contrast, K12 protein expression was most pronounced in cells subcultivated in limbal CM, but markedly weaker in both corneal CM and in 3T3 CM (Fig. 5E–5H).

To verify the immunocytochemical findings, mRNA and protein expression of K10, K12, and, additionally, Pax6 in response to different CM were analyzed by real-time RT-PCR and Western blotting. Consistently, K10 mRNA expression was reduced in limbal CM (about fivefold; *p* < .05) and increased in both corneal CM compared with 3T3 CM (Fig. 5I). In contrast, the K12 mRNA expression level was significantly increased in limbal CM (about fivefold; *p* < .005) compared with the other CM derived from corneal and 3T3 fibroblasts (Fig. 5J). Concordantly, Pax6 expression was most pronounced in limbal CM, whereas it was hardly detectable in 3T3 CM (Fig. 5K). Western blot analysis confirmed the highest expression levels of K12 and Pax6 protein in cells cultivated in limbal CM, whereas K10 protein expression was the lowest under these conditions (Fig. 5L, 5M). 3T3 CM was found to suppress expression of both K12 and Pax6, but to induce expression of K10 (Fig. 5L, 5M).

**Figure 6:**
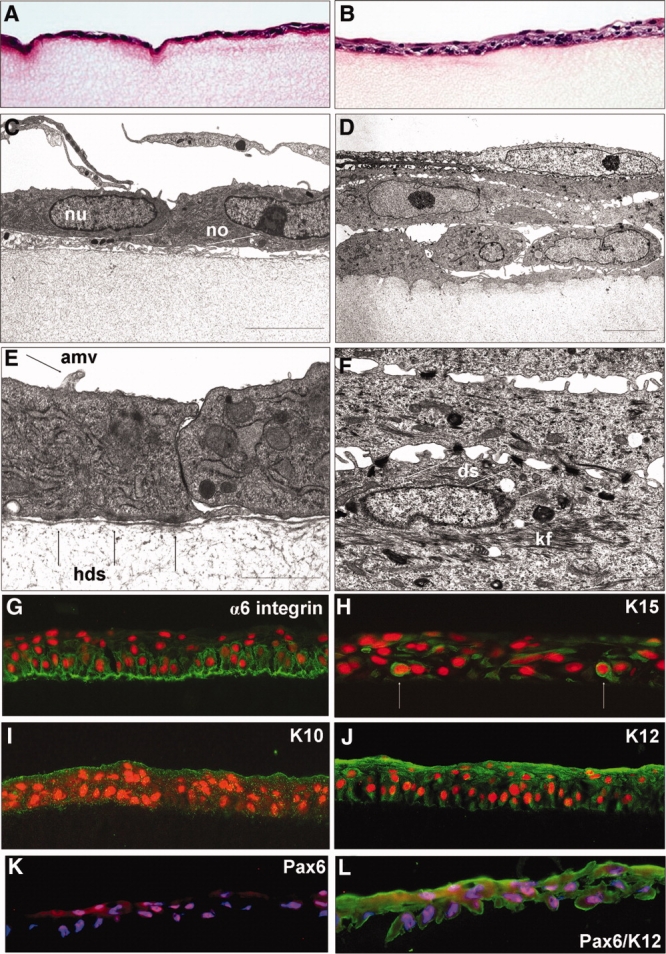
Phenotypic appearance of cell sheets subcultivated in limbal fibroblast conditioned medium on fibrin gels coated with laminin-5. **(A, C, E):** Light and electron microscopic appearance of a two-layered epithelial cell sheet after 1 week of culture. **(B, D, F):** Light and electron microscopic appearance of a multilayered cell sheet after 2 weeks of culture. The cells show ultrastructural signs of epithelial differentiation such as apical microvilli, keratin filaments, desmosomes, and hemidesmosome. **(G):** Expression of α6 integrin (green fluorescence), a putative stem and progenitor cell marker, in the basal cell layer of the epithelial sheet. **(H):** Expression of K15 (green fluorescence), a putative stem cell marker, in a few single basal cells of the epithelial sheet (arrows). **(I):** Expression of K10 (green fluorescence), a marker of epidermal differentiation, in the superficial cell layer. **(J):** Expression of K12 (green fluorescence), a marker of corneal differentiation, throughout all epithelial cell layers. **(K):** Expression of Pax6 (red fluorescence), a transcription factor for K12, in nuclei of epithelial cells. **(L):** Colocalization of K12 (green) and Pax6 (red) expression in epithelial cells. Magnification: 40× **(A, B)**, 100× **(G, I, J, K)**, 200× **(H, L).** Scale bars, 5 μm **(C, D)** and 1 μm **(E, F)**. Nuclear staining was performed with propidium iodide (red) or 4′,6-diamidino-2-phenylindole (blue). Abbreviations: amv, apical microvilli; ds, desmosomes; hds, hemidesmosomes; kf, keratin filaments; no, nucleolus; nu, nucleus.

For light and electron microscopic analysis of cell sheets, clonal cells were subcultivated on fibrin gels coated with laminin-5 in different CM. By light microscopy, both limbal and 3T3 CM were confirmed to produce regular cell sheets composed of small epitheloid cells arranged in one or two layers after 8-10 days (Fig. 6A) and three to five layers after 14-16 days (Fig. 6B) of culture. Transmission electron microscopy revealed cuboid to elongated cells containing euchromatin-rich nuclei with prominent nucleoli and showing ultrastructural signs of epithelial differentiation, such as apical microvilli, keratin filaments, hemidesmosomes, and desmosomes between adjacent cells, already apparent after 1 week of culture (Fig. 6C, 6E) and more pronounced after a prolonged culture time (Fig. 6D, 6F).

Immunohistological analysis revealed that stratified epithelial cell sheets cultured in CM from limbal fibroblasts expressed α6 integrin in all cells and K15 in single cells of the basal cell layer (Fig. 6G, 6H), indicating preservation of stem and progenitor cells in culture. Weak expression of K10 was confined to the superficial cell layer (Fig. 6I), whereas K12 expression was more prominent throughout all epithelial cell layers (Fig. 6J). The transcription factor Pax6 was immunolocalized to nuclei of most epithelial cells throughout all cell layers (Fig. 6K) and colocalized with K12 (Fig. 6L).

Collectively, these data indicate that the phenotype and differentiation of HF epithelial stem cells can be influenced by a specific microenvironment and that factors derived from limbal fibroblasts appear to induce expression of the corneal epithelial differentiation marker K12 together with its transcription factor Pax6, whereas factors derived from corneal fibroblasts rather promote expression of the epidermal differentiation marker K10.

## DISCUSSION

Reconstruction of the stratified ocular surface epithelium in patients with bilateral limbal stem cell deficiency is one of the most challenging problems in clinical ophthalmology. In order to replenish the stem cell pool, it is clearly desirable to use autologous cells for ex vivo culture, tissue engineering, and transplantation, as this avoids the risk of allogenic immune rejection and the need for immunosuppression. A major strategy is based on autologous stem cells taken from stratified epithelia of other areas of the body. Recent progress in this field suggests that oral mucosal epithelium [8–10], conjunctival epithelium [33, 34], and epidermis [35, 36] may serve as alternative sources of autologous adult stem cells, which can be used to reconstruct the ocular surface in animal models and patients with limbal stem cell deficiency. However, insufficient clinical long-term outcomes justify a continued search for new autologous stem cell sources.

The HF and its connective tissue sheath contain several, not yet completely defined, stem cell populations with multipotent capacities [19]. Mesenchymal and nestin-expressing cells in the HF bulge region have been shown to be able to differentiate into neuronal, glial, smooth muscle, adipocytic, melanocytic, and other phenotypes in vitro [37–39]. In addition, the HF represents a major repository of multipotent keratinocyte stem cells in both mouse and human skin, which have the potential to differentiate into HF, sebaceous gland, and epidermis [40]. It has been even suggested that the HF bulge area contains the ultimate stem cell of the entire epidermis/HF compartment [17, 32, 41]. The potential of HF stem cells to differentiate into corneal epithelial cells has, however, not been investigated so far.

A comparison of the corneal epithelium and the follicular epithelium reveals that keratinocyte stem cells of these two systems share several features, such as expression of K15, K19, p63, α6 integrin, and β1 integrin [17]. Both stem cell populations reside in special microenvironments involving the basement membrane components type IV collagen and laminin-5 [5] as well as close spatial association with neighboring mesenchymal cells. Consistently, it has been demonstrated that corneal epithelium can be reprogrammed to form epidermis and HF by dermal developmental signals in tissue recombination experiments [42, 43]. These observations provided the rationale for the present study, presuming that HF epithelial stem cells might in turn be able to transdifferentiate into a corneal epithelial phenotype in response to corneal- or limbus-specific microenvironmental conditions. These microenvironmental conditions have been partially replicated in vitro using characteristic extracellular matrix components and fibroblast conditioned media containing specific soluble factors and signaling molecules.

The findings of the present study confirmed that holoclone-forming adult stem cells are clustered in the bulge region of the murine vibrissa HF [44, 45] and that bulge stem cells can be effectively isolated both by mechanical dissection/enzymatic dissociation and FACS using α6 integrin labeling. The findings provided first-time evidence that epithelial HF stem cells can transdifferentiate into a corneal epithelial-like cell lineage after clonal enrichment on a feeder layer and subcultivation under conditions mimicking the limbal rather than the corneal microenvironment. In response to laminin-5, elevated extracellular calcium concentration, and limbal fibroblast CM, HF keratinocytes expressed significantly higher mRNA and protein levels of K12, a marker of terminally differentiated corneal epithelium, as compared with CM derived from corneal fibroblasts or 3T3 fibroblasts. In contrast, expression of K10, a marker of terminally differentiated epidermal keratinocytes, was significantly downregulated by limbal fibroblast CM as compared with the other CM. Upregulation of K12 by limbal CM was paralleled by an induction of Pax6 mRNA and protein. The paired box gene six (*Pax6*), which is the universal master control gene for eye morphogenesis, is postnatally expressed in the corneal epithelium, where it maintains the normal epithelial phenotype and acts as a transcription factor for *K12* gene expression [46, 47]. The concomitant induction of K12 and Pax6 by limbal CM provides conclusive evidence of the transdifferentiation potential of HF stem cells into a corneal epithelial phenotype. The stratified cell sheets cultivated in limbal CM displayed morphological characteristics of corneal epithelial cells and structural integrity, as indicated by the presence of desmosomes and hemidesmosomes. They could be established on fibrin gels, which represent ideal carriers for future transplantation purposes of corneal epithelial-like cell sheets due to their standardized, noninfectious, biodegradable, and adhesive properties.

The present findings confirm the importance of the limbal stem cell niche, which normally regulates the behavior and differentiation of limbal stem and progenitor cells, for inducing transdifferentiation into a corneal epithelial-like phenotype. They also suggest that replication of niche factors in vitro increases the efficiency and lineage determination of stem cell-based cultivation methods. Previous studies using the inductive properties of niches to direct the differentiation of stem cells into the desired epidermal and corneal epithelial lineages focused primarily on mouse embryonic stem cells [48–51]. These were either cocultivated with stromal fibroblasts [49], cultivated with specific matrix components and fibroblast-conditioned media [48, 52], or injected into an appropriate environment in animal models in vivo [50, 51]. Expression levels of K1/K10 and K3/K12 were used as an indication of differentiation into an epidermal or corneal epithelial lineage, respectively. Those studies demonstrated that corneal epithelial-like cells can be obtained from embryonic stem cells, if they are given limbus-specific differentiation signals and environmental factors, such as type IV collagen and limbal fibroblast CM [52]. Similarly, epidermal stem cells obtained from adult Rhesus monkeys were demonstrated to transdifferentiate into corneal epithelial-like cells when cocultured with human corneal limbal stroma and corneal epithelial cells [35].

The present findings provide further evidence that soluble and matrix-associated limbal niche factors, that is, CM derived from stromal fibroblasts and laminin-5, can also induce transdifferentiation of murine vibrissa HF-derived adult stem cells into a corneal epithelial-like phenotype, as indicated by upregulation of K12/Pax6 expression and cellular morphology. Although these findings need further substantiation using in vivo functional studies in animal models, they provide the first step towards the design of protocols to use human autologous HF stem cells for replacement of the corneal epithelium in therapeutic applications. Important parallels between mouse and human HF bulge cells validate the use of the mouse as a model for future investigations on human HF epithelial stem cells. Due to their multipotency, easy accessibility, and high proliferation rate ex vivo, these represent an attractive source of autologous adult stem cells and a promising therapeutic tool for ocular surface reconstruction and restoration of visual function in patients with ocular surface disorders.
